# Supplementary material for: Blood gas and lactate analysis in nesting loggerhead (Caretta caretta) and green (Chelonia mydas) sea turtles from southeastern Florida, USA
Source: PLoS One. 2026 Mar 2;21(3):e0343737. doi: 10.1371/journal.pone.0343737 (PMC12952625; doi:10.1371/journal.pone.0343737)
Supplement: Attachment — Submitted filename: EY Review_UPDATE_231125.docx [file pone.0343737.s001.docx]

Summary of the Manuscript

This manuscript presents blood gas and lactate measurements from nesting loggerhead and green turtles using two point-of-care devices. The authors analyse the data separately by species, comparing loggerheads and green turtles and then examining body-size relationships, analyte correlations, agreement between devices, and temporal patterns within each species (with significant size relationships and seasonal patterns observed in loggerheads). The resulting species-specific reference intervals are clinically and ecologically useful and help fill an important gap, particularly for field teams working with nesting turtles.

Overall Recommendation

Major Revision.

The study is valuable, but key parts of the manuscript need clearer explanation, more cautious interpretation, and some restructuring. A number of the physiological claims go a bit beyond what the data can actually support, and several methodological pieces need better transparency. The Discussion is also quite long and at times feels like a literature review that does not always relate back to the data, and is sometimes quite speculative.

General Comments

Several parts of the manuscript would benefit from clearer explanation and slightly more cautious interpretation of the results. First, the sample sizes need to be made more transparent. While 49 loggerheads and 30 green turtles were initially sampled, the final analyte-specific numbers are noticeably lower (e.g., loggerheads n = 43–44; greens n = 21). It would help readers if the authors clarified that only turtles with usable blood values were included for each analyte, and briefly explained why the numbers vary across variables (e.g., incomplete cartridges, missing analytes, sample-quality issues). This is particularly important because the reference intervals and species comparisons are based entirely on these final datasets.

A second major point relates to the repeated use of phrases such as “across the nesting season.” The current sampling structure doesn’t quite support this wording. Loggerheads were sampled across the full June–August 2021 period, but green turtles were sampled across two different years, with the 2023 individuals coming only from mid- to late season. As a result, the temporal coverage isn’t parallel between species, which could affect the ability to detect any seasonal patterns in green turtles. It’s also unclear whether loggerheads and greens have the same nesting phenology at this site, or how the timing of sampling aligns with each species’ natural nesting peak. Only the loggerhead temporal analysis is shown (Fig. 3); the green turtle regressions are not presented, even though they are described as non-significant. Given this uneven temporal coverage and the mixing of years, the statements about physiological patterns “across the nesting season,” and the idea that species differ in their seasonal physiological strategies, feel a bit stronger than the data can support. A more cautious phrasing here, along with a brief acknowledgement of these sampling limitations and potential inter-annual differences, would help readers interpret the findings more accurately.

There are also several behavioural and reproductive explanations—such as differences in clutch size, number of eggs laid, digging effort, duration on the beach, or pauses during nest construction—that are used to interpret physiological differences between species. However, none of these behavioural variables were measured in this study. It also isn’t shown whether clutch size actually differs between species in this dataset. More importantly, the physiological patterns don’t line up neatly with these proposed explanations (for example, loggerheads had higher pO₂, while greens had higher pCO₂ and bicarbonate). Since these behaviours weren’t quantified and the proposed mechanisms aren’t directly supported by the measured variables, I think these interpretations need to be presented as speculative rather than definitive.

Finally, parts of the Discussion feel quite long and occasionally drift into general literature review or physiological speculation that doesn’t always tie back to the actual findings. Tightening up some of these sections, keeping the focus on what was actually measured, and making sure interpretations remain grounded in the presented data would strengthen the manuscript considerably. There are also a few places where terminology—especially “nesting season” versus “season”—isn’t used consistently, and some key terms aren’t defined. A quick cleanup of terminology would improve clarity, particularly where temporal interpretations are involved.

In addition, the statistical methods section would benefit from a bit more clarity around how normality was assessed, how choices were made between parametric and non-parametric tests, and how outliers and reference-interval methods were handled. Some of this information appears later in the Results, but including a brief explanation in the Methods would help with overall transparency (see Methods and Results for more detail).

In addition, a light editorial pass would be helpful. Some sentences, especially in the Introduction, Methods and Discussion, are quite long and rely heavily on parentheses, which can interrupt the flow. It may also help to standardise terminology and abbreviations (e.g., “point-of-care,” “POC,” “Point-of-Care”; “i-STAT,” “i-STAT 1,” “i-STAT one”) and to use one consistent form throughout the text, tables and figure captions.

Specific Comments

ABSTRACT

L26–27: Sample size clarification as above — it should be clear that only turtles with usable analytes were included, and the final n varies.

L29–30: Recommend replacing “nest deposition” with “oviposition,” which is the more standard term for sea turtle egg-laying.

L26–30: Because all analyses in the manuscript are species-specific (reference intervals, correlations, morphometric effects, temporal analyses, agreement analyses), it would help readers if this structure were made clearer earlier in the manuscript, including here in the Abstract and in the Methods.

L36–37: Consider grouping all loggerhead-specific results together (including the emergence-to-sampling time) and then presenting the green turtle results. This would improve flow and make the structure easier for readers to follow.

L39–41: This conclusion may be overstated as written. The green turtle dataset is smaller, uneven across years, and the temporal analysis was non-significant, making it hard to infer species-level differences in seasonal physiological strategy. The loggerhead seasonal trends were statistically significant but very weak (r² only 0.09–0.21), suggesting that the actual effect may be small. A more cautious phrasing may be more appropriate here.

L41–43: The phrase “offer temporo-spatial comparisons” reads as though the study conducted temporal and spatial comparisons, but no spatial analyses were done, and the only temporal analysis shown was for loggerheads. If the intention is simply to state that the data could support temporo-spatial work in the future, that should be made clearer. As written, the wording is ambiguous and slightly overstates what was actually analysed.

INTRODUCTION

General

Some parts of the Introduction go into quite detailed general sea turtle biology (e.g., clutch sizes, number of clutches per season, long migrations, and global nesting comparisons with places like Oman and Cape Verde, capital breeding, opportunistic feeding, vitellogenesis, migration costs, annual vs. biennial nesting). While interesting, these details aren’t used in the study and don’t directly set up the physiological questions being addressed. The Introduction would read more clearly if it focused on the physiological context and why the Florida nesting population provides a good opportunity for this work. Streamlining the broader life-history material would help keep the framing tighter and more relevant.

L63-67: As mentioned above, this section would be improved by focusing on the broader energetic demands of migration and nesting, without the detailed life-history values.

L77–91: The physiological context here is very useful, but the detailed behavioural description of nesting (duration, site investigation, digging sequence) may be more than needed, since these behaviours were not measured in this study. Streamlining would help maintain focus.

L106–108: This is a useful point, but the mention of “inter-species differences” seems like a separate idea and might be clearer as its own sentence.

L108-114: As mentioned previously, as most of the analyses are carried out separately for each species, it may be worth mentioning it here as well.

L111-112: Again recommend replacing “nest deposition” with oviposition here and throughout the manuscript.

ETHICS STATEMENT

L119–120: The wording “standard veterinary protocols and practices” feels quite general. It could be helpful to mention which specific guidelines or institutional protocols were followed, or to give a citation, so the ethics statement is a bit more transparent.

METHODS

L122–126: The sentence describing the ATV patrols is quite dense—it combines species, locations, coordinates, and two separate patrol periods into a single line, which makes it difficult to follow. Breaking this into two shorter sentences would make the sampling scheme much clearer.

In addition, the timing of sampling would benefit from clarification. Both species were sampled from June–August 2021, with an additional small group of green turtles sampled mid-season in 2023. It may also help to note that Juno and Jupiter Beaches are adjacent and effectively represent one continuous nesting area.

See General Comments for the broader issues regarding temporal coverage, nesting phenology, and the interpretation of “across the nesting season.”

L128–129: It is unclear whether turtles missing identification received both an external flipper tag and a PIT tag, or only one of these. The phrase “and/or” combined with “these tags were placed” makes the tagging procedure ambiguous. Clarifying whether both tag types were applied when no tags were present would improve clarity.

L129-131: The statement that turtles were approached once 50–75% of the clutch had been deposited is clear, but it would help to briefly explain how this proportion was estimated. For example, were specific behavioural cues used, or is this based on an established field procedure for determining when it is safe to approach a nesting female? A short clarification (or a citation, if this timing follows a standard nesting-monitoring practice) would strengthen the reproducibility of this step.

L131-133: The description of the health assessment feels a bit too broad at the moment. Since the Results later refer to turtles being “clinically normal,” having “adequate body condition,” “normal behaviour,” and various external injuries, it would help readers if the Methods gave a little more detail about what those assessments actually involved. For example, what did the physical exam include? How was body condition evaluated? Were injuries classified in a particular way? And what criteria were used to judge mentation or behaviour? Even a short outline, or a reference to an established assessment approach if one was followed, would make this section clearer and more reproducible.

L134: The wording “flexible tape measures” is a bit vague. It also sounds like more than one tape was used, which may not be what you meant. If a specific type of tape was used—like the fibreglass measuring tapes that are standard for curved carapace measurements—it would be helpful to just say that.

L134: Write out CCW in full first time mentioned

L135–136: The phrase “logistical challenges prevented collection…” is quite broad and leaves the reader guessing. If you’re able to briefly indicate the type of issue (e.g., limited time with the animal, access constraints, or field conditions), it would make the limitation clearer. If the details truly can’t be specified, the current wording is still acceptable—but a little more context would help.

L140-141: It would be helpful to briefly mention how sample quality was checked (e.g., haemolysis, clots etc.)

L141: Recommend adding “of collection” (i.e., “within 60 s of collection”).

L143–146: The wording could be tightened; as written, this sentence implies that the CG4+ cartridges apply only to the directly measured analytes and not the calculated ones. Please revise for clarity.

L146–147: The phrasing here is awkward, especially “as entered by the user.” A cleaner explanation of how temperature correction is applied would make this easier to follow.

L147–149: Multiple references are cited for the temperature-correction and calculated analyte methods. These references use different formulas in the literature. Recommend the authors check that all cited references are consistent with the specific calculations used in this study and revise accordingly if needed.

L150: Suggest replacing “used” with “obtained” (“could not be obtained due to permit restrictions…”).

L150-151: Please confirm that the references cited support the use of environmental (air) temperature for temperature correction. For example, McNally et al. validated surface temperature as a surrogate for cloacal temperature, whereas other studies emphasize obtaining internal body temperature whenever possible. Clarifying the rationale for using air temperature here would be helpful. This is also a study limitation.

L152–154: The description of the pH and bicarbonate calculations is hard to follow. It may help to clarify that the >25°C correction was applied across the board, and ensure the cited references align with the approach used.

L154–157: This sentence reads more like interpretation than methods. It may need rewording, and any interpretive comments could be moved out of the Methods.

L157–159: The timing of the Nova Lactate Plus measurement isn’t clear. Please state roughly how soon after running the CG4+ cartridge the Nova reading was taken.

L174: Please clarify that the paired samples for the t-test came from the same turtles.

L163–178: The statistical methods section would benefit from a bit more detail. It would be helpful for the authors to briefly explain why the selected reference-interval method was chosen and how they assessed whether it was appropriate for the data (e.g., distribution, sample size, outliers). Clarifying whether normality was checked before using parametric tests (Pearson correlations, t-tests, linear regressions) would also improve readability, along with how decisions were made between standard and Welch’s t-tests. A short explanation of how outliers and Box–Cox transformations were handled, and whether paired-difference distributions were checked for the Bland–Altman analysis, would round out this section and make the workflow easier to follow.

L167–170: It’s unclear which predictor variables were paired with which outcomes, and whether regressions were run separately for each species. A clearer description of the regression setup would help.

L171–172: This sentence ends with “or” and seems incomplete. Please clarify how species comparisons were done and how decisions between t-test types were made.

L173–178: The workflow for the method-comparison analyses is difficult to follow. A short clarification of the order and purpose of Passing–Bablok, Bland–Altman, paired t-tests, and Deming regression would make this section much clearer.

RESULTS
L184–185: This reads more like a Methods detail than a Result. You could move the reasoning for increasing sample size to the Methods and simply report that 30 green turtles were included here.

L185–187: One loggerhead was sampled twice, but it isn’t clear how this was handled statistically. This should be clarified in the Methods.

L188-189: The sentence about injuries is a bit unclear. Rephrasing it or giving a little more context would help.

L190–194: The description of injuries is difficult to follow, and the use of “stage 4” and “stage 5” wound healing based on a thesis reference may not be widely understood by readers. Briefly describing the staging system or rephrasing using more standard clinical terminology would improve clarity. Additionally, the sentence is long and contains multiple injury descriptions, which could be reorganized for readability.

L195: “All green turtles showed evidence of mating scars” could use a bit more detail about what was classified as a mating scar and whether they were fresh or healed.

L196–201: This long sentence combines multiple injuries and individuals, which makes it difficult to read. Breaking it up would help. The healing-stage terminology also may need brief clarification.

L202–203: The Results describe turtles as clinically normal with adequate body condition, but the Methods don’t describe how these assessments were done. A short outline of the health assessment criteria in the Methods would make this clearer.

L207–208: The sentence “Only samples from the first encounter… per turtle” belongs in the Methods rather than the Results. Please move.

L211–216: The explanation for excluding base excess, sO₂, and TCO₂ is methodological and should also be moved to the Methods section.

Table 1: The note “Parametric or robust methods for sample sizes 20 ≤ x < 120…” is better placed in the Methods. It is also unclear how the original sample sizes (49 loggerheads; 30 greens) reduced to the numbers in the table. Please clarify.

Table 2: Table 2 mainly summarises previously published data. You may want to place this in Supplementary Materials. Since there are many numerical values and correction notes, it would also be good to re-check all entries against the original papers—I noticed at least one discrepancy (e.g., SE USA 2001, time to analysis). Listing sample sizes (n) for each row or sampling condition would also improve clarity.

Table 3: The caption states that both Spearman and Pearson correlations were used, but the table shows only one correlation per comparison, which appears to be Spearman. Please clarify which test was used and ensure consistency with the Methods. The Methods should also briefly describe how you decided between Spearman and Pearson (Spearman not currently mentioned).

Species Comparisons

It is unclear how the sample size dropped from 49 loggerheads and 30 greens to the numbers used in the reference intervals (e.g., 43–44 loggerheads; 21 greens) and the species-comparison tests (e.g., df = 62). Please clarify which samples were excluded, why, and how final sample sizes were determined. You may also want to streamline repeated reporting of mean ± SD across Table 1, Table 2, and the text.

Figure 1: Figure 1 closely duplicates the detailed species-comparison statistics already provided in the text. Consider simplifying the figure or reducing repeated statistics in the text. Also, the pO₂ and pCO₂ panels look inconsistent with the ranges reported in the Results—please check axis labels and plotted values.

Morphometrics

The phrase “significantly increased but was weakly associated” is awkward. You may want to rephrase as “a weak but statistically significant association.”

Figure 2: Fig. 2 repeats the numerical results already presented in the text (slope, r², P, N). Consider simplifying the figure or reducing duplication.

Clutch deposition date

Recommend updating the heading to Oviposition date.

You already note that the loggerhead relationships are weak; a brief comment on what these very low r² values mean for the seasonal interpretation would help keep the message aligned with the data.

Figure 3: As with other figures, the statistical details in the caption and text are repeated. Consider streamlining either the figure or the accompanying text.

Duration of emergence to sampling

Suggested clearer wording: “Time from emergence to blood collection in loggerheads ranged from 22 to 73 min (mean ± SD = 43 ± 11 min) and was not significantly related to blood gases or lactate (all P > 0.050).”

Lactate method comparison

The text states that conversion equations were generated, but it’s unclear whether these came from the Passing–Bablok regressions (Fig. 4a,d) or from the Deming regression described in the Methods. Please clarify which method produced the conversion formulas.

Figure 4: The panel order (a, d, b, e, c, f) is difficult to follow; reordering or relabelling would help. There is extensive repetition of the statistical results already described in the text. The caption is very long and includes methodological explanations better suited for the Methods. Device terminology is inconsistent (“i-STAT,” “i-STAT one,” “i-STAT 1”). Please standardise.

DISCUSSION

The statement about being the first study to examine these analytes “across the course of the nesting season” is broadly correct, but you might consider adding a short qualifier, as the seasonal trends were only significant for loggerheads and the green turtle data were more limited. This would help reflect the structure of the dataset.

REFERENCE INTERVALS

This section goes well beyond interpreting the reference intervals and becomes difficult to follow in places. A number of paragraphs move into detailed physiology, hormonal regulation, nesting behaviour, or comparisons with unrelated capture conditions, without clearly tying these points back to the results from this dataset. Streamlining this material so it focuses on what directly informs interpretation of the new reference intervals would improve clarity.

Several detailed physiological explanations seem to follow interpretations from Wallace et al. (2008), but the primary citation (Jackson & Prange, 1979) does not fully support some of the mechanistic conclusions drawn. Re-phrasing these parts more cautiously would help avoid overstating the evidence.

The discussion of “life-stage classes” could also be clearer. Some examples (e.g., entangled turtles, directly captured turtles) describe capture conditions rather than true life stages, and revising this terminology would improve accuracy.

There are also several long comparisons of blood-gas and lactate values across many unrelated studies. Condensing these comparisons, and linking them more directly to the present dataset, would make the narrative easier to follow. Additionally, the interpretation of lactate differences appears reversed: lower lactate usually reflects less exertion, not more. Revising this would make the physiological interpretation more accurate.

The point linking lower lactate to handling stress may also need adjustment. Since lactate mainly reflects muscular exertion, and the turtles were described as not struggling, it may be better to state that the lower values are consistent with minimal exertion and possibly lower handling-related stress, rather than definitive evidence of no stress.

One practical clarification could also help: blood sampling during oviposition can sometimes cause turtles to pause laying. If this occurred, noting it (and whether it influenced sampling duration or analyte values) would be useful. If it was never observed, a brief statement confirming that sampling did not interrupt oviposition would reassure readers that the behaviour was not disrupted.

Overall, this section would benefit from tightening and focusing on the most relevant physiological context, specifically, the patterns found in this study. A more concise approach will keep the reader centred on the new reference intervals and the key results, without losing important context.

CORRELATIONS BETWEEN BLOOD GASES AND LACTATE and SPECIES DIFFERENCES

The discussion around correlations and species differences contains several interpretations that reach beyond what the data can directly support. In both sections, a number of physiological mechanisms are assigned to the observed patterns in lactate and blood gases—such as aerobic capacity, anaerobic thresholds, oxygen storage, metabolic rate, and nesting effort—even though none of these variables were measured in this study. Blood gases and lactate can suggest general acid–base dynamics, but they cannot on their own confirm detailed metabolic strategies, so some of the mechanistic explanations should be framed more cautiously.

Similarly, behavioural descriptions (e.g., nest-digging effort, time spent on the beach, frequency of pauses) are used to infer underlying metabolic processes, but these behaviours were not quantified here. Without behavioural or metabolic measurements, it is difficult to link species differences in lactate or pH directly to differences in aerobic capacity or fuel use. Interpretations such as similar lactate indicating higher aerobic capacity in green turtles should therefore be softened, as lactate alone is not a definitive indicator of aerobic strategy.

A few of the citations also appear to be stretched beyond what they support. For example, Jackson & Prange (1979) did not study nesting green turtles, and metabolic scope data from hatchlings cannot be extrapolated to adults during nesting. Some statements linking turtle size, oxygen stores, and metabolic rate may also simplify relationships that were not assessed in this dataset.

Overall, both sections would benefit from reducing speculative or mechanistic explanations and keeping the focus on what the data clearly show: the observed correlations among analytes and the measured species differences in blood gas and lactate values. Presenting the interpretations more cautiously, and ensuring that all supporting references align directly with the statements made, would strengthen the clarity and accuracy of the discussion

LACTATE METHOD COMPARISONS

The manuscript notes that an i-STAT cartridge costs “~$25 USD” and a Nova Lactate Plus strip costs “~$2 USD.” Because prices vary by region and tend to change over time, it may be clearer to describe these simply as relative consumable costs rather than providing specific dollar amounts.

Some of the wording around device “validation” may also be a bit strong. The analyses performed constitute a method comparison rather than full analytical validation (e.g., accuracy, precision, repeatability), so a small adjustment in terminology would help keep expectations aligned with what was actually done.

The detailed temperature-operating ranges for each device may be more specific than necessary unless those limits directly affected data interpretation in this study. More importantly, because temperature corrections were applied to the blood gas values, it would be helpful for the manuscript to report the actual environmental temperatures recorded during sampling. Blood gas and lactate measurements in ectotherms are temperature-sensitive, so noting the temperature range used for corrections would improve clarity for readers.

CONCLUSION

A few points in the conclusion could be phrased more cautiously to stay aligned with the data. The study did include a seasonal analysis, but only loggerheads showed significant trends—and these were weak (r² = 0.09–0.21). Green turtle data were limited and uneven across years, so wording such as “across the nesting season” may overstate what can be concluded. A softer phrasing would help.

Similarly, the suggestion that the two species use different physiological strategies during high-energy nesting is stronger than the results can support, as these mechanisms were not directly measured. Presenting this instead as a possible explanation would be more appropriate.

The closing section on reference intervals could also be streamlined. Terms like “species-specific,” “cohort-specific,” and “within a given species” overlap and make the message unclear. A simpler statement—e.g., that species-specific RIs will assist future comparisons—would read more cleanly. Additionally, the term “cohort-specific” may not be the best fit here, as it’s not clear what cohort refers to in this context.

Finally, the sentence about identifying stressors or disease isn’t supported by the analyses presented, as the study did not evaluate disease or clinical diagnostic use. Consider softening or removing this unless additional evidence is provided.

Editor Comments

The manuscript is scientifically sound and clinically useful, but several sentences in the Methods (and to a lesser extent the Introduction and Discussion) are quite awkwardly phrased or dense. I have flagged some examples in the line-by-line section, but have avoided doing full copy-editing. A general language and style review (especially for long, parenthetical sentences and consistency of terminology) would improve readability.
