# Supplementary material for: Blood gas and lactate analysis in nesting loggerhead (Caretta caretta) and green (Chelonia mydas) sea turtles from southeastern Florida, USA
Source: PLoS One. 2026 Mar 2;21(3):e0343737. doi: 10.1371/journal.pone.0343737 (PMC12952625; doi:10.1371/journal.pone.0343737)
Supplement: Attachment — Submitted filename: Response to reviewers_01.07.2026.docx [file pone.0343737.s003.docx]

January 8, 2026

Dear Editor and Reviewers,

Please find enclosed our revised manuscript entitled “Blood gas and lactate analysis in nesting loggerhead (*Caretta caretta*) and green (*Chelonia mydas*) sea turtles from southeastern Florida, USA” for consideration of publication as a research article in Plos ONE. We collectively thank you for your constructive, insightful and well-reasoned feedback of our manuscript.

We have provided two revised versions of the manuscript, one showing track changes and one with these changes accepted. Additionally, we have replied individually to each point raised by the reviewers; please see responses below. Line numbers refer to the track changes version when “all markup” is selected for review (please note: the line numbering does appear non-continuous unless “simple” or “no markup” is selected due to a limitation with Word itself, so the line numbers are unfortunately inconsistent).

In sum, multiple changes have been made throughout the manuscript to address the reviewers’ feedback, and the abstract has also been revised accordingly. Of particular importance, we have clarified some of the methodology and results, including sample collection across portions of the nesting season, turtle handling, physical examination, sample quality control, temperature-correction of blood gases, and statistical analyses. Moreover, we have reorganized and consolidated the discussion section, including removal of subheaders, to better integrate and hopefully present a clearer analysis of the data to the reader. As suggested by one reviewer, we have also clarified terminology and edited wording such that the discussion of species differences in regard to metabolic capacity are presented in a more presumptive manner.

Thank you again for your time, feedback, and consideration of this manuscript. Thank you for your continued considering our manuscript for publication. We look forward to hearing from you soon.

Sincerely,

Faye E. Giebink, Justin R. Perrault, Madison Toonder, Sarah E. Hirsch, Derek M. Aoki, Craig A. Harms, Charles J. Innis, and Nicole I. Stacy

**Reviewer #1:**

This is a very well written article with valuable information regarding the effects of life stage and species on blood gas and lactate measurements in sea turtles. There are a few small areas that I believe could be clarified.

**Response:** Thank you for your positive feedback and review of our manuscript.

**1.** You state that the animals were less likely to have stress as a factor affecting blood sampling for these animals as they were in the nesting fixed action pattern. Did you measure the length of time the animals were handled prior to blood draw? Previous data shows that even 15 minutes of handling can significantly alter blood gas and lactate levels so time that your animals were handled seems relevant.

**Response:** Thank you for these considerations. We did not measure the exact length of time the animals were handled prior to blood draw. However, the entire process of the physical exam, morphometric measurements, and venipuncture were generally under 15 minutes. The cited study (Mones et al. 2021) showing significant acidosis and hyperlactatemia in turtles within 15 minutes was performed on “normal” “awake” turtles (i.e., not turtles that are in a nesting fixed action pattern, which is a sort-of “trance” in which nesting turtles are minimally responsive to external stimuli during oviposition, as was done for our study). Given this difference in the physiologic state of turtles between studies, it makes more exact comparison regarding the length of time that turtles were handled difficult.

**2.** Fig 1 - pO2 was significantly different in loggerheads and green turtles - I don't see that this was discussed anywhere. Venous pO2 does not correlate with arterial pO2 and is generally thought to be not clinically relevant. Therefore, there is not much to discuss but if it is in a figure, it should be discussed at least briefly.

**Response:** Thank you for making this observation! This has been added to the discussion (p. 22; lines 1053-1117): “Green turtles may therefore require a longer overall timeframe for nesting activities, with intermittent periods of rest during longer crawls towards the dunes as compared to loggerheads with preference for nesting mid beach, to balance aerobic demands and compensate for metabolic acidosis. These considerations may also explain the higher pCO_2_ and lower pO_2_ in green turtles compared to loggerheads.”

**3.** P22 end of paragraph correlations between blood gas and lactate (for some reason line numbering does not extend past p11) - you state the acidotic effects are counteracted by neutralization of bicarbonate through lactic acid. Is this true? Generally metabolic acid base compensation is regulated by bicarbonate metabolism through the kidneys. I am not aware of lactic acid being part of this - is this a species difference or is it not known?

**Response:** Yes, this is correct. The neutralization of lactic acid by bicarbonate is a phenomenon that occurs during anaerobic metabolism in both humans and other animals. There are multiple mechanisms by which the body achieves acid-base homeostasis: (1) neutralization via buffer systems (both intracellular and extracellular), (2) exhalation by the respiratory system, and (3) clearance by the renal system. (Shaw and Gregory 2022 succinctly reviews the physiology of acid-base balance: <https://www.sciencedirect.com/science/article/pii/S2058534922000816>). During strenuous exercise, for example, lactic acid accumulates in the skeletal muscle and rapidly dissociates into lactate and hydrogen ions, which decrease pH; bicarbonate ions in the blood then bind hydrogen to form carbonic acid and pH is increased. Since the wording of this sentence might have been confusing, we have changed the wording to (p. 19; lines 866-868): “These acidotic effects are presumably counteracted by the compensatory mechanisms of respiratory alkalosis through hyperventilation and neutralization of lactic acid by bicarbonate.” We have also corrected the issue regarding line numbering; however, **please note:** the line numbering does appear non-continuous unless “simple” or “no markup” is selected due to a limitation with Word itself, so the line numbers are unfortunately inconsistent.

**Reviewer #2:**

Summary of the Manuscript:

This manuscript presents blood gas and lactate measurements from nesting loggerhead and green turtles using two point-of-care devices. The authors analyze the data separately by species, comparing loggerheads and green turtles and then examining body-size relationships, analyte correlations, agreement between devices, and temporal patterns within each species (with significant size relationships and seasonal patterns observed in loggerheads). The resulting species-specific reference intervals are clinically and ecologically useful and help fill an important gap, particularly for field teams working with nesting turtles.

Overall Recommendation:

Major Revision. The study is valuable, but key parts of the manuscript need clearer explanation, more cautious interpretation, and some restructuring. A number of the physiological claims go a bit beyond what the data can actually support, and several methodological pieces need better transparency. The Discussion is also quite long and at times feels like a literature review that does not always relate back to the data and is sometimes quite speculative.

**Response:** Thank you for your constructive feedback! As per your detailed comments below, we have revised the entire manuscript, particularly to clarify methodology, consolidate the discussion section, and overall changed some wording to hopefully make things more understandable to the reader. Given the concerns regarding the discussion, we have also adjusted the wording in several sentences to lessen the “definitiveness” of the conclusions and ensure the reader understands that discussion of species differences is presumptive given the current data and warrants further study with larger sample sizes.

General Comments:

**1.** Several parts of the manuscript would benefit from clearer explanation and slightly more cautious interpretation of the results. First, the sample sizes need to be made more transparent. While 49 loggerheads and 30 green turtles were initially sampled, the final analyte-specific numbers are noticeably lower (e.g., loggerheads n = 43–44; greens n = 21). It would help readers if the authors clarified that only turtles with usable blood values were included for each analyte, and briefly explained why the numbers vary across variables (e.g., incomplete cartridges, missing analytes, sample-quality issues). This is particularly important because the reference intervals and species comparisons are based entirely on these final datasets.

**Response:** Thank you for this suggestion. This has been clarified in results (p. 10; lines 453-456): “Due to logistical challenges with i-STAT and/or Nova Lactate Plus POC devices in the field, blood samples obtained from some turtles were unable to be analyzed by either one or both instruments, resulting in varying sample sizes between analyzers.”

2. A second major point relates to the repeated use of phrases such as “across the nesting season.” The current sampling structure doesn’t quite support this wording. Loggerheads were sampled across the full June–August 2021 period, but green turtles were sampled across two different years, with the 2023 individuals coming only from mid- to late season. As a result, the temporal coverage isn’t parallel between species, which could affect the ability to detect any seasonal patterns in green turtles. It’s also unclear whether loggerheads and greens have the same nesting phenology at this site, or how the timing of sampling aligns with each species’ natural nesting peak. Only the loggerhead temporal analysis is shown (Fig. 3); the green turtle regressions are not presented, even though they are described as non-significant. Given this uneven temporal coverage and the mixing of years, the statements about physiological patterns “across the nesting season,” and the idea that species differ in their seasonal physiological strategies, feel a bit stronger than the data can support. A more cautious phrasing here, along with a brief acknowledgement of these sampling limitations and potential inter-annual differences, would help readers interpret the findings more accurately.

**Response:** Thank you for your suggestion. We have changed the wording “across portions of the nesting season” to qualify this concern. We have also changed some other wording to indicate that the relationship is weak and also added discussion of this possible limitation to the end of the discussions section (p. 24; lines 1345-1351): “While turtles would ideally have been sampled across the entire nesting season (late April to early September and May to late September for loggerheads and green turtles, respectively), both species were still sampled across 70 days, with an additional 24 days for green turtles, during the peak nesting period, which provides valuable data for temporal correlations. However, the smaller sample size in green turtles reduces statistical power and thereby limits the possibility for more conclusive interpretations regarding differing strategies to manage reproduction between both species.”

3. There are also several behavioural and reproductive explanations—such as differences in clutch size, number of eggs laid, digging effort, duration on the beach, or pauses during nest construction—that are used to interpret physiological differences between species. However, none of these behavioural variables were measured in this study. It also isn’t shown whether clutch size actually differs between species in this dataset. More importantly, the physiological patterns don’t line up neatly with these proposed explanations (for example, loggerheads had higher pO₂, while greens had higher pCO₂ and bicarbonate). Since these behaviours weren’t quantified and the proposed mechanisms aren’t directly supported by the measured variables, I think these interpretations need to be presented as speculative rather than definitive.

**Response:** Thank you for these considerations, but we respectfully disagree with this feedback since the behavioral and reproductive aspects are well described in the literature and consistent by species. Therefore, measuring the behavioral variables and differences between species would have been beyond the scope of this study and not needed. The same applies for clutch sizes and other end points; nevertheless, we do not make any interpretations regarding any possible effects of clutch size or number of eggs laid on species differences in blood gases or lactate. The behavioral interpretations are speculative as we wish to present the reader with multiple possible interpretative explanations for the differences noted between species. As suggested by the reviewer, we have therefore edited some of the wording to make these claims less definitive (e.g., using words such as “may” – p. 22, lines 1051, 1059; or “could” – p. 24, line 1341, etc).

4. Finally, parts of the Discussion feel quite long and occasionally drift into general literature review or physiological speculation that doesn’t always tie back to the actual findings. Tightening up some of these sections, keeping the focus on what was actually measured, and making sure interpretations remain grounded in the presented data would strengthen the manuscript considerably. There are also a few places where terminology—especially “nesting season” versus “season”—isn’t used consistently, and some key terms aren’t defined. A quick cleanup of terminology would improve clarity, particularly where temporal interpretations are involved.

**Response:** Thank you for your feedback. We have addressed this throughout the discussion. As suggested, we have also changed the wording “across portions of the nesting season” to qualify this concern.

5. In addition, the statistical methods section would benefit from a bit more clarity around how normality was assessed, how choices were made between parametric and non-parametric tests, and how outliers and reference-interval methods were handled. Some of this information appears later in the Results, but including a brief explanation in the Methods would help with overall transparency (see Methods and Results for more detail).

**Response:** Thank you for your feedback. The statistical methods section has been clarified as suggested here and, in more detail, below – for example (p. 9, lines 375-382): “For each species, Pearson correlations were used for normally distributed variables (using Shapiro-Wilk tests and Q-Q plots), and Spearman correlations were applied otherwise; no outliers were identified. Ordinary least-squares regression analysis was used to determine associations of blood gas and lactate concentrations with CCL_min_ and with nest deposition date for both loggerheads and green turtles and, in loggerheads, with time from emergence to blood sampling. Species comparisons for blood gas and lactate concentrations were made using independent samples or Welch’s t-tests for each turtle.”

6. In addition, a light editorial pass would be helpful. Some sentences, especially in the Introduction, Methods and Discussion, are quite long and rely heavily on parentheses, which can interrupt the flow. It may also help to standardise terminology and abbreviations (e.g., “point-of-care,” “POC,” “Point-of-Care”; “i-STAT,” “i-STAT 1,” “i-STAT one”) and to use one consistent form throughout the text, tables and figure captions.

**Response:** Edited as suggested. These terms have been standardized throughout the manuscript.

Specific Comments:

ABSTRACT

L26–27: Sample size clarification as above — it should be clear that only turtles with usable analytes were included, and the final n varies.

**Response:** Edited as suggested. This has been clarified in results (p. 10, lines 453-456): “Due to logistical challenges with i-STAT and/or Nova Lactate Plus POC devices in the field, blood samples obtained from some turtles were unable to be analyzed by either one or both instruments, resulting in varying sample sizes between analyzers” and the abstract has been reworded. Due to word count limitations for the abstract, an explanation of the variance in sample size was not added here; however, this has now been included in detail in the manuscript.

L29–30: Recommend replacing “nest deposition” with “oviposition,” which is the more standard term for sea turtle egg-laying.

**Response:** The authors prefer the term “nest deposition” as we feel this is more encompassing of the full nesting process (emerging, searching for the right spot, digging, laying the full clutch of eggs, covering the nest, etc), as opposed to “oviposition” which is more specific to the act of laying the egg(s) itself. We have standardized this term throughout the text for consistency.

L26–30: Because all analyses in the manuscript are species-specific (reference intervals, correlations, morphometric effects, temporal analyses, agreement analyses), it would help readers if this structure were made clearer earlier in the manuscript, including here in the Abstract and in the Methods.

**Response:** Added as suggested. We have added clarifying words such as “species-specific” and “for each species” both in the abstract as well as the main text (e.g., p. 2, lines 28-30; p. 6, lines 187-189).

L36–37: Consider grouping all loggerhead-specific results together (including the emergence-to-sampling time) and then presenting the green turtle results. This would improve flow and make the structure easier for readers to follow.

**Response:** Thank you for this suggestion. We have edited this as suggested (p. 2, lines 36-39): “In loggerheads, lactate positively correlated with curved carapace length, while pH declined and lactate increased across the nesting season, and there were no relationships between blood analytes and time from emergence to blood collection. No morphometric or seasonal trends were observed for green turtles.”

L39–41: This conclusion may be overstated as written. The green turtle dataset is smaller, uneven across years, and the temporal analysis was non-significant, making it hard to infer species-level differences in seasonal physiological strategy. The loggerhead seasonal trends were statistically significant but very weak (r² only 0.09–0.21), suggesting that the actual effect may be small. A more cautious phrasing may be more appropriate here.

**Response:** Edited as suggested. We adjusted the phrasing for more cautious interpretation: “mild trend,” and “suggests” in addition to adding the qualification “across portions of the nesting season” (p. 2, lines 41-43).

L41–43: The phrase “offer temporo-spatial comparisons” reads as though the study conducted temporal and spatial comparisons, but no spatial analyses were done, and the only temporal analysis shown was for loggerheads. If the intention is simply to state that the data could support temporo-spatial work in the future, that should be made clearer. As written, the wording is ambiguous and slightly overstates what was actually analysed.

**Response:** Done. We have removed this wording.

INTRODUCTION

General

Some parts of the Introduction go into quite detailed general sea turtle biology (e.g., clutch sizes, number of clutches per season, long migrations, and global nesting comparisons with places like Oman and Cape Verde, capital breeding, opportunistic feeding, vitellogenesis, migration costs, annual vs. biennial nesting). While interesting, these details aren’t used in the study and don’t directly set up the physiological questions being addressed. The Introduction would read more clearly if it focused on the physiological context and why the Florida nesting population provides a good opportunity for this work. Streamlining the broader life-history material would help keep the framing tighter and more relevant.

**Response:** Thand you for these thoughts, but we respectfully disagree that the details provided in the introduction are irrelevant to the study. First, the explanation of this specific location in Florida as a globally and regionally important aggregation for nesting turtles of both species tells the reader why the sample population used in this study is important. Elucidating the major threats, including the threats to sea turtle nesting grounds, underscores the importance of studying nesting turtles specifically. Second, the next two paragraphs detail the high energy demands undergone by nesting turtles which spurred the interest in evaluating blood gases and lactate for this cohort (i.e., nesting females) and become directly relevant to the discussion/analysis of the data. We more broadly explain the energetic demands that go into reproduction as a whole during each nesting season (hence the detailed description of breeding migration, number of clutches, clutch sizes, capital breeding/feeding strategies). Then, since turtles deposit multiple clutches within a single season, we also explain why each individual nesting event is so energetically taxing to the turtle in and of itself. A few sentences have been reorganized in the introduction to try to make this clearer to the reader.

L63-67: As mentioned above, this section would be improved by focusing on the broader energetic demands of migration and nesting, without the detailed life-history values.

**Response:** Thank you for your thoughts. Please see response above.

L77–91: The physiological context here is very useful, but the detailed behavioural description of nesting (duration, site investigation, digging sequence) may be more than needed, since these behaviours were not measured in this study. Streamlining would help maintain focus.

**Response:** Please see response above. We believe these details become relevant to the discussion since they are important to support the interpretative considerations.

L106–108: This is a useful point, but the mention of “inter-species differences” seems like a separate idea and might be clearer as its own sentence.

**Response:** Edited as suggested. The sentence has been split up and the meaning of “associated inter-species differences” has been reworded for clarification (p. 5, lines 173-178): “However, blood gas and lactate reference intervals have yet to be established for any nesting loggerhead or green turtle population, nor have these analytes been examined across the course of the nesting season. These data could provide critical insight into the expenditure of energy stores and metabolic changes during reproduction and elucidate associated differences in physiologic strategies in nesting females between species.”

L108-114: As mentioned previously, as most of the analyses are carried out separately for each species, it may be worth mentioning it here as well.

**Response:** Edited as suggested. As in the abstract, we have added clarifying words such as “species-specific” and “for each species” etc.

L111-112: Again, recommend replacing “nest deposition” with oviposition here and throughout the manuscript.

**Response:** Please see previous response (abstract section). We prefer the term “nest deposition” as we feel this is more encompassing of the full nesting process and have standardized this term throughout the text for consistency.

ETHICS STATEMENT

L119–120: The wording “standard veterinary protocols and practices” feels quite general. It could be helpful to mention which specific guidelines or institutional protocols were followed, or to give a citation, so the ethics statement is a bit more transparent.

**Response:** Edited. We have adjusted the wording (p. 6, lines 197-198): “All handling and sampling procedures were consistent with standard veterinary protocols and best practices for sea turtles” and added the pertaining reference:

McMichael EA, Hapeman A, Stacy B, Stokes L, Seminoff J, Haas, H, Martin S, Sasso C, Hargrove S, Frutchey K. National sea turtle research standard methods manual. US Department of Commerce, NOAA Technical Memorandum. 2025; NMFS-F/SPO-262.

METHODS

L122–126: The sentence describing the ATV patrols is quite dense—it combines species, locations, coordinates, and two separate patrol periods into a single line, which makes it difficult to follow. Breaking this into two shorter sentences would make the sampling scheme much clearer.

**Response:** Thank you for this suggestion. We have split these sentences and reworded for clarification (please see next response below).

In addition, the timing of sampling would benefit from clarification. Both species were sampled from June–August 2021, with an additional small group of green turtles sampled mid-season in 2023. It may also help to note that Juno and Jupiter Beaches are adjacent and effectively represent one continuous nesting area.

**Response:** Edited as suggested, We have reworded these sentences for clarification (pg. 6, lines 200-206): “All-terrain vehicles were used to conduct annual nightly patrols on adjacent Juno and Jupiter Beaches in Palm Beach County, Florida (26.943°N, -80.072°W to 26.837°N, -80.014°W) from late March through August of each year. Samples were opportunistically collected from nesting loggerhead and green turtles as they were encountered across the nesting season. Both species were sampled from 4 June to 13 August 2021, with additional green turtles sampled from 10 July to 3 August 2023 to increase sample size for the generation of reference intervals.”

See General Comments for the broader issues regarding temporal coverage, nesting phenology, and the interpretation of “across the nesting season.”

**Response:** We have addressed this suggestion and added the qualifier “across portions of the nesting season” and also elucidated in the methods that the beaches were patrolled late March through August, and that samples were opportunistically collected from turtles as they were encountered during the patrolled portion of the nesting season (please see response above). As suggested, this limitation has also been added to the end of the discussion (p. 24, lines 1334-1340): “While turtles would ideally have been sampled across the entire nesting season (late April to early September and May to late September for loggerheads and green turtles, respectively), both species were still sampled across 70 days, with an additional 24 days for green turtles, during the peak nesting period, providing valuable data for temporal correlations. However, the smaller sample size in green turtles reduces statistical power and thereby limits the possibility for more conclusive interpretations regarding differing physiologic strategies to manage reproduction between both species.”

L128–129: It is unclear whether turtles missing identification received both an external flipper tag and a PIT tag, or only one of these. The phrase “and/or” combined with “these tags were placed” makes the tagging procedure ambiguous. Clarifying whether both tag types were applied when no tags were present would improve clarity.

**Response:** Thank you for the suggestion. This has been edited (pg. 7, lines 224-225): “If either type of tag was not present, the tag was placed according to standard protocols.”

L129-131: The statement that turtles were approached once 50–75% of the clutch had been deposited is clear, but it would help to briefly explain how this proportion was estimated. For example, were specific behavioural cues used, or is this based on an established field procedure for determining when it is safe to approach a nesting female? A short clarification (or a citation, if this timing follows a standard nesting-monitoring practice) would strengthen the reproducibility of this step.

**Response:** Edited. This has been changed to “Turtles were approached during their nesting fixed action pattern once at least 50 eggs had been deposited” (p. 6, lines 206-207).

L131-133: The description of the health assessment feels a bit too broad at the moment. Since the Results later refer to turtles being “clinically normal,” having “adequate body condition,” “normal behaviour,” and various external injuries, it would help readers if the Methods gave a little more detail about what those assessments actually involved. For example, what did the physical exam include? How was body condition evaluated? Were injuries classified in a particular way? And what criteria were used to judge mentation or behaviour? Even a short outline, or a reference to an established assessment approach if one was followed, would make this section clearer and more reproducible.

**Response:** These explanations have been added to the methods (pg. 7, lines 228-231): “Body condition was based on gross observation of neck and shoulder fat thickness, and external injuries were characterized as previously described for loggerhead turtles, with mating scars classified by the presence of fresh scratches or scars on the shoulder(s) and/or cranial carapace.”

The following references have also been cited:

Page-Karjian A, Chabot R, Stacy NI, Morgan AS, Valverde RA, Stewart S, et al. Comprehensive health assessment of green turtles Chelonia mydas nesting in southeastern Florida, USA. Endanger Species Res. 2020;42: 21–35. doi:10.3354/ESR01036

Ataman A, Gainsbury AM, Manire CA, Hoffmann SL, Page-Karjian A, Hirsch SE, et al. Evaluating prevalence of external injuries on nesting loggerhead sea turtles Caretta caretta in southeastern Florida, USA. Endanger Species Res. 2021;46: 137–146. doi:10.3354/esr01149

Klingshirn S. Injury analysis of leatherback sea turtles (*Dermochelya coriacea*) nesting on northern Palm Beach County, Florida, USA beaches. M.Sc. Thesis, Florida Atlantic University. 2021.

L134: The wording “flexible tape measures” is a bit vague. It also sounds like more than one tape was used, which may not be what you meant. If a specific type of tape was used—like the fibreglass measuring tapes that are standard for curved carapace measurements—it would be helpful to just say that.

**Response:** A generic vinyl tape measure purchased from Amazon was used in this study. This has now been addressed (p. 7, line 231): “Flexible vinyl tape measures were used to determine minimum and standard curved carapace length (CCL_min_ and CCL, respectively) and curved carapace width (CCW).”

L134: Write out CCW in full the first time it is mentioned.

**Response:** Thank you for noting this. Edited (p. 7, line 233; please see response above).

L135–136: The phrase “logistical challenges prevented collection…” is quite broad and leaves the reader guessing. If you’re able to briefly indicate the type of issue (e.g., limited time with the animal, access constraints, or field conditions), it would make the limitation clearer. If the details truly can’t be specified, the current wording is still acceptable—but a little more context would help.

**Response:** Addressed (p. 7, lines 234-236): “Logistical challenges prevented extended monitoring for the collection of time from emergence to blood draw in green turtles, which exhibit significantly longer nesting stage durations than loggerheads and spend up to 2 hours body pitting and digging before oviposition.”

L140-141: It would be helpful to briefly mention how sample quality was checked (e.g., haemolysis, clots etc.)

**Response:** Thank you for your suggestion. This has been added to the methods (p. 7-8, lines 242-293): “The remaining sample volume was simultaneously analyzed using a Nova Lactate Plus POC analyzer (Nova® Biomedical, Waltham, Massachusetts USA). Blood samples were evaluated prior to analysis and excluded if plasma had greater than 1+ hemolysis or lipemia following subsequent processing in the laboratory.”

L141: Recommend adding “of collection” (i.e., “within 60 s of collection”).

**Response:** Done.

L143–146: The wording could be tightened; as written, this sentence implies that the CG4+ cartridges apply only to the directly measured analytes and not the calculated ones. Please revise for clarity.

**Response:** Thank you for your feedback. The wording has been revised for clarity (p.8, lines 294-299): “The i-STAT CG4+ cartridges directly measure pH, partial pressure of carbon dioxide (pCO_2_), partial pressure of oxygen (pO_2_), and lactate, then calculate base excess, bicarbonate (HCO_3_), total carbon dioxide (TCO_2_), and oxygen saturation (sO_2_) using standard equations. Since pH, pO_2_, and pCO_2_ are temperature-dependent analytes, the i-STAT analyzes blood at 37°C. The user may then “correct” these values for body temperatures other than 37°C by entering a different temperature into the analyzer.”

L146–147: The phrasing here is awkward, especially “as entered by the user.” A cleaner explanation of how temperature correction is applied would make this easier to follow. **Response:** This has been edited as suggested (p. 8, lines 298-299): “The user may then “correct” these values for body temperatures other than 37°C by entering a different temperature into the analyzer.”

L147–149: Multiple references are cited for the temperature-correction and calculated analyte methods. These references use different formulas in the literature. Recommend the authors check that all cited references are consistent with the specific calculations used in this study and revise accordingly if needed.

**Response:** Done. We have checked all the references and reformatted/reworded some of this section to hopefully make the reason for the selection of equations clearer. We have also added the exact calculations used so the reader is not confused (please see p. 8-9, lines 301-363).

L150: Suggest replacing “used” with “obtained” (“could not be obtained due to permit restrictions…”).

**Response:** Done.

L150-151: Please confirm that the references cited support the use of environmental (air) temperature for temperature correction. For example, McNally et al. validated surface temperature as a surrogate for cloacal temperature, whereas other studies emphasize obtaining internal body temperature whenever possible. Clarifying the rationale for using air temperature here would be helpful. This is also a study limitation.

**Response:** The references cited for the equations use temperature-correction of blood gases in sea turtles regardless of which temperature (cloacal, surface, or air) since these subtle differences would result in minimal/not clinically relevant differences in blood gases. The reason we used environmental temperature is due to permit restrictions, which did not allow for us to obtain cloacal temperature. The cited literature which was included for the support of environmental temperature as a surrogate for body temperature are Stacy et al. 2017 and Adamovicz et al. 2018 (p. 8, line 301).

L152–154: The description of the pH and bicarbonate calculations is hard to follow. It may help to clarify that the >25°C correction was applied across the board, and ensure the cited references align with the approach used.

**Response:** Done. We have reviewed the references, and the wording has been clarified (p. 8, lines 306-311): “While empirical decline in the slope of pH below 25°C in green sea turtles has prompted other studies to use different calculations for pH correction at these body temperatures, comparison of previously derived formulae for pH for temperatures >25°C and <25°C revealed that there were no significant differences (<0.5%) in pH between equations for turtles our study. Thus, pH was calculated using the standard equation for body temperatures >25°C for all turtles regardless of environmental temperature.”

L154–157: This sentence reads more like interpretation than methods. It may need rewording, and any interpretive comments could be moved out of the Methods.

**Response:** We have elected to keep this in methods but have reworded to clarify why it is relevant in regard to methodology, since it informed which calculation(s) would be used for temperature correction (please also see responses above) (p. 8, lines 306-311): “While empirical decline in the slope of pH below 25°C in green sea turtles has prompted other studies to use different calculations for pH correction at these body temperatures, comparison of previously derived formulae for pH for temperatures >25°C and <25°C revealed that there were no significant differences (<0.5%) in pH between equations for turtles our study. Thus, pH was calculated using the standard equation for body temperatures >25°C for all turtles regardless of environmental temperature.”

L157–159: The timing of the Nova Lactate Plus measurement isn’t clear. Please state roughly how soon after running the CG4+ cartridge the Nova reading was taken.

**Response:** Edited. They were analyzed simultaneously, immediately (within 60 sec) of blood collection. This has been clarified (p. 7-8, lines 240-291): “Blood from the syringes was immediately (i.e., within 60 s of collection) placed into CG4+ cartridges and analyzed using a VetScan i-STAT POC device (Abbott Point of Care Inc., Princeton, New Jersey USA). The remaining sample volume was simultaneously analyzed using a Nova Lactate Plus POC analyzer (Nova^®^ Biomedical, Waltham, Massachusetts USA).”

L174: Please clarify that the paired samples for the t-test came from the same turtles.

**Response:** Edited (p. 9, lines 381-382): “Species comparisons for blood gas and lactate concentrations were made using independent samples or Welch’s t-tests for each turtle.”

L163–178: The statistical methods section would benefit from a bit more detail. It would be helpful for the authors to briefly explain why the selected reference-interval method was chosen and how they assessed whether it was appropriate for the data (e.g., distribution, sample size, outliers). Clarifying whether normality was checked before using parametric tests (Pearson correlations, t-tests, linear regressions) would also improve readability, along with how decisions were made between standard and Welch’s t-tests. A short explanation of how outliers and Box–Cox transformations were handled, and whether paired-difference distributions were checked for the Bland–Altman analysis, would round out this section and make the workflow easier to follow.

**Response:** As stated in the second sentence, statistical analysis is based on ASVCP guidelines and readers may refer to this if they have any questions regarding why certain tests/analyses were used.

References:

Friedrichs KR, Harr KE, Freeman KP, Szladovits B, Walton RM, Barnhart KF, et al. ASVCP reference interval guidelines: Determination of de novo reference intervals in veterinary species and other related topics. Vet Clin Pathol. 2012;41: 441–453. doi:10.1111/vcp.12006

Le Boedec K. Reference interval estimation of small sample sizes: a methodologic comparison using a computer-simulation study. Vet Clin Pathol. 2019;48: 335–346. doi:10.1111/vcp.12725

L167–170: It’s unclear which predictor variables were paired with which outcomes, and whether regressions were run separately for each species. A clearer description of the regression setup would help.

**Response:** We have clarified the wording by adding “for each species” (p. 9, line 375). As stated in the second sentence, statistical analysis is based on ASVCP guidelines and readers may refer to this if they have any questions regarding why certain tests/analyses were use (see above: Friedrichs et al. 2012, Le Boedec 2019).

L171–172: This sentence ends with “or” and seems incomplete. Please clarify how species comparisons were done and how decisions between t-test types were made.

**Response:** As stated in the second sentence, statistical analysis is based on ASVCP guidelines and readers may refer to this if they have any questions regarding why certain tests/analyses were used (see above: Friedrichs et al. 2012, Le Boedec 2019).

L173–178: The workflow for the method-comparison analyses is difficult to follow. A short clarification of the order and purpose of Passing–Bablok, Bland–Altman, paired t-tests, and Deming regression would make this section much clearer.

**Response:** As stated in the second sentence, statistical analysis is based on ASVCP guidelines and readers may refer to this if they have any questions regarding why certain tests/analyses were used (see above: Friedrichs et al. 2012, Le Boedec 2019).

RESULTS
L184–185: This reads more like a Methods detail than a Result. You could move the reasoning for increasing sample size to the Methods and simply report that 30 green turtles were included here.

**Response:** Edited (p. 10, lines 448-449): “In total, 49 nesting loggerheads and 30 nesting green turtles were sampled from 4 June to 13 August 2021 and 10 July to 3 August 2023.”

L185–187: One loggerhead was sampled twice, but it isn’t clear how this was handled statistically. This should be clarified in the Methods.

**Response:** Edited. We have clarified this in the methods (p. 7, lines 220-221): “For the establishment of reference intervals, only samples from the first encounter were included (i.e., one sample per turtle).”

L188-189: The sentence about injuries is a bit unclear. Rephrasing it or giving a little more context would help.

**Response:** Done. We have reworded and consolidated this section (p. 10, lines 465-472): “On visual examination, all turtles were determined to be in good body condition, exhibited normal behavior and mentation, and had no observable evidence of overt traumatic injury or disease (e.g. fibropapillomatosis). Mild external injuries were noted on some individuals.”

L190–194: The description of injuries is difficult to follow, and the use of “stage 4” and “stage 5” wound healing based on a thesis reference may not be widely understood by readers. Briefly describing the staging system or rephrasing using more standard clinical terminology would improve clarity. Additionally, the sentence is long and contains multiple injury descriptions, which could be reorganized for readability.

**Response:** Done. We have reworded and consolidated this section and removed the use of the stages of wound healing to make it less convoluted for the reader (p. 10, lines 472-523): “Three loggerheads had nearly or completely healed carapacial injuries, likely from interaction with boat propellers, and one had minor (<5% missing) healed injuries to both hind limbs. Two loggerheads had healed amputations involving approximately 50% of a the right forelimb or the right hindlimb, and another had a small, circular, partially healed wound on its neck that was approximately 5 cm deep. All green turtles showed evidence of mating scars from interactions with males. Three green turtles had carapacial scabbing or healed injuries from boating interactions, and three others had healed amputations involving approximately 20–50% of the right hind limbs. One of the turtles also had small injuries to the caudal carapace and evidence of constriction from fishing line entanglement to the right hind limb that had since healed. Given that any injuries were mild or incidental, and turtles were otherwise clinically normal, these were deemed unlikely to affect blood gas or lactate measurements.”

L195: “All green turtles showed evidence of mating scars” could use a bit more detail about what was classified as a mating scar and whether they were fresh or healed.

**Response:** Done. An explanation of how mating scars were identified has been added to the methods section (pg. 7, line 231): “… mating scars classified by the presence of fresh scratches or scars on the shoulder(s) and/or cranial carapace” including the associated reference:

Page-Karjian A, Chabot R, Stacy NI, Morgan AS, Valverde RA, Stewart S, et al. Comprehensive health assessment of green turtles Chelonia mydas nesting in southeastern Florida, USA. Endanger Species Res. 2020;42: 21–35. doi:10.3354/ESR01036

L196–201: This long sentence combines multiple injuries and individuals, which makes it difficult to read. Breaking it up would help. The healing-stage terminology also may need brief clarification.

**Response:** Thank you for your suggestion. We have reworded and consolidated this section and removed the use of the stages of wound healing to make it less convoluted for the reader: (p. 10, lines 465-523): “On visual examination, all turtles were determined to be in good body condition, exhibited normal behavior and mentation, and had no observable evidence of overt traumatic injury or disease (e.g. fibropapillomatosis). Mild external injuries were noted on some individuals. Three loggerheads had nearly or completely healed carapacial injuries, likely from interaction with boat propellers, and one had minor (<5% missing) healed injuries to both hind limbs. Two loggerheads had healed amputations involving approximately 50% of a single limb, and another had a small, circular, partially healed wound on its neck that was approximately 5 cm deep. All green turtles showed evidence of mating scars from interactions with males. Three green turtles had carapacial scabbing or healed injuries from boating interactions, and three others had healed amputations involving approximately 20–50% of the right hind limbs. One of the turtles also had small injuries to the caudal carapace and evidence of constriction from fishing line entanglement to the right hind limb that had since healed. Given that any injuries were mild or incidental, and turtles were otherwise clinically normal, these were deemed unlikely to affect blood gas or lactate measurements.”

L202–203: The Results describe turtles as clinically normal with adequate body condition, but the Methods don’t describe how these assessments were done. A short outline of the health assessment criteria in the Methods would make this clearer.

**Response:** Done. An explanation of how body condition is evaluated in turtles has been added to the methods section (p. 7, lines 228-229): “Body condition was based on gross observation of neck and shoulder fat thickness” with the associated reference:

Page-Karjian A, Chabot R, Stacy NI, Morgan AS, Valverde RA, Stewart S, et al. Comprehensive health assessment of green turtles Chelonia mydas nesting in southeastern Florida, USA. Endanger Species Res. 2020;42: 21–35. doi:10.3354/ESR01036

L207–208: The sentence “Only samples from the first encounter… per turtle” belongs in the Methods rather than the Results. Please move.

**Response:** Done (p. 6, lines 207-208): “No turtles were tagged or handled before eggs were laid, and sampling did not disrupt oviposition for any of the turtles in this study.”

L211–216: The explanation for excluding base excess, sO₂, and TCO₂ is methodological and should also be moved to the Methods section.

**Response:** Done (p. 9, lines 365-370): “Data for base excess, sO_2_, and TCO_2_ were excluded from this study because i-STAT determination of base excess assumes standard human hemoglobin and plasma protein concentrations, sO_2_ is related to species-specific distinctions in blood oxygen affinity that differ between sea turtles and mammals, between sea turtle species, and between age classes of the same sea turtle species, and TCO_2_ is based on formulae for mammals and clinically interchangeable with pCO_2_.”

Table 1: The note “Parametric or robust methods for sample sizes 20 ≤ x < 120…” is better placed in the Methods. It is also unclear how the original sample sizes (49 loggerheads; 30 greens) reduced to the numbers in the table. Please clarify.

**Response:** As stated in the methods, statistical analysis is based on ASVCP guidelines which outlines when parametric or robust methods should be used based on sample size. The authors would prefer to keep this note in the table caption since the righthand column lists the analytes that were used for reference interval establishment as per these guidelines. We also believe that some readers look at the table alone, without referencing the text, which may warrant keeping the brief explanation in the caption.

Table 2: Table 2 mainly summarises previously published data. You may want to place this in Supplementary Materials. Since there are many numerical values and correction notes, it would also be good to re-check all entries against the original papers—I noticed at least one discrepancy (e.g., SE USA 2001, time to analysis). Listing sample sizes (n) for each row or sampling condition would also improve clarity.

**Response:** Thank you for this consideration. The authors prefer this table within the main portion of the manuscript, as we feel it is important for the discussion regarding comparison of blood gases and lactate data between different life-class stages, etc. Regarding the concern for discrepancy, we have reviewed the sources and confirmed that the time to analysis for the Harms et al. 2003 (SE USA 2001) study is 10 minutes.

Table 3: The caption states that both Spearman and Pearson correlations were used, but the table shows only one correlation per comparison, which appears to be Spearman. Please clarify which test was used and ensure consistency with the Methods. The Methods should also briefly describe how you decided between Spearman and Pearson (Spearman not currently mentioned). **Response:** Thank you for noting this. This has been clarified in both the caption and in the methods section (p. 9, lines 375-377): “For each species, Pearson correlations were used for normally distributed variables (using Shapiro-Wilk tests and Q-Q plots), and Spearman correlations were applied otherwise; no outliers were identified.”

Species Comparisons

It is unclear how the sample size dropped from 49 loggerheads and 30 greens to the numbers used in the reference intervals (e.g., 43–44 loggerheads; 21 greens) and the species-comparison tests (e.g., df = 62). Please clarify which samples were excluded, why, and how final sample sizes were determined. You may also want to streamline repeated reporting of mean ± SD across Table 1, Table 2, and the text.

**Response:** Edited. At the beginning of the results section, we have clarified (p. 10, lines 465-468): “Due to logistical challenges with i-STAT and/or Nova Lactate Plus POC devices in the field, blood samples obtained from some turtles were unable to be analyzed by either one or both instruments, resulting in varying sample sizes between analyzers.”

Figure 1: Figure 1 closely duplicates the detailed species-comparison statistics already provided in the text. Consider simplifying the figure or reducing repeated statistics in the text. Also, the pO₂ and pCO₂ panels look inconsistent with the ranges reported in the Results—please check axis labels and plotted values.

**Response:** Done. We have removed repeated statistics from the figures and elected to keep them in the text instead. Thank you also for noting the inconsistency regarding the pO2 and pCO2 – this has been removed from the figure and fixed in the text.

Morphometrics

The phrase “significantly increased but was weakly associated” is awkward. You may want to rephrase as “a weak but statistically significant association.”

**Response:** Edited (p. 16, lines 671-672): “Lactate as determined by i-STAT showed a weak but statistically significant association with increasing CCL_min_ in loggerheads.”

Figure 2: Fig. 2 repeats the numerical results already presented in the text (slope, r², P, N). Consider simplifying the figure or reducing duplication.

**Response:** Done. We have removed these numerical results from all the figures and kept them in the manuscript text.

Clutch deposition date

Recommend updating the heading to Oviposition date.

**Response:** The authors prefer the term “nest deposition” as we feel this is more encompassing of the full nesting process (emerging, searching for the right spot, digging, laying the full clutch of eggs, covering the nest, etc), as opposed to “oviposition” which is more specific to the act of laying the egg(s) itself. We have standardized this term throughout the text for consistency.

You already note that the loggerhead relationships are weak; a brief comment on what these very low r² values mean for the seasonal interpretation would help keep the message aligned with the data.

**Response:** Edited. We have changed some wording to indicate that the relationship is weak and also added discussion of this possible limitation to the end of the discussion section (p. 24, lines 1354-1360): “While turtles would ideally have been sampled across the entire nesting season (late April to early September and May to late September for loggerheads and green turtles, respectively), both species were still sampled across 70 days, with an additional 24 days for green turtles, during the peak nesting period, providing valuable data for temporal correlations. However, the smaller sample size in green turtles reduces statistical power and thereby limits the possibility for more conclusive interpretations regarding differing physiologic strategies to manage reproduction between both species.”

Figure 3: As with other figures, the statistical details in the caption and text are repeated. Consider streamlining either the figure or the accompanying text.

**Response:** Done. We have removed these numerical results from all the figures and kept them in the manuscript text.

Duration of emergence to sampling

Suggested clearer wording: “Time from emergence to blood collection in loggerheads ranged from 22 to 73 min (mean ± SD = 43 ± 11 min) and was not significantly related to blood gases or lactate (all P > 0.050).”

**Response:** Edited as per suggestion (p. 17, lines 712-713): “Time from emergence to blood collection in loggerheads ranged from 22 to 73 min (mean ± SD = 43 ± 11 min) and was not significantly related to blood gases or lactate (all P > 0.050).”

Lactate method comparison

The text states that conversion equations were generated, but it’s unclear whether these came from the Passing–Bablok regressions (Fig. 4a,d) or from the Deming regression described in the Methods. Please clarify which method produced the conversion formulas.

**Response:** Done. Both the methods and the discussion state that the conversion equation was generated from Deming regression; we have repeated this again here in the results for clarity.

Figure 4: The panel order (a, d, b, e, c, f) is difficult to follow; reordering or relabelling would help. There is extensive repetition of the statistical results already described in the text. The caption is very long and includes methodological explanations better suited for the Methods. Device terminology is inconsistent (“i-STAT,” “i-STAT one,” “i-STAT 1”). Please standardise. **Response:** The authors believe it is important to have repetition of certain critical analyses in both the panel and the text since many readers read tables or figures as stand-alone table or figure without taking the time to refer back to the text itself. We also believe that the panel order makes sense when visually looking at the figure with panels being sorted horizontally by species and vertically by type of analysis. Additionally, we have fixed the terminology regarding the i-STAT and Nova Lactate Plus devices so it is standardized throughout the manuscript.

DISCUSSION

The statement about being the first study to examine these analytes “across the course of the nesting season” is broadly correct, but you might consider adding a short qualifier, as the seasonal trends were only significant for loggerheads and the green turtle data were more limited. This would help reflect the structure of the dataset.

**Response:** Thank you for your suggestion. We have changed the wording “across portions of the nesting season” to qualify this concern. We have also changed some other wording to indicate that the relationship is weak and also added discussion of this possible limitation to the end of the discussions section (p. 24; lines 1362-1368): “While turtles would ideally have been sampled across the entire nesting season (late April to early September and May to late September for loggerheads and green turtles, respectively), both species were still sampled across 70 days, with an additional 24 days for green turtles, during the peak nesting period, which provides valuable data for temporal correlations. However, the smaller sample size in green turtles reduces statistical power and thereby limits the possibility for more conclusive interpretations regarding differing strategies to manage reproduction between both species.”

REFERENCE INTERVALS

This section goes well beyond interpreting the reference intervals and becomes difficult to follow in places. A number of paragraphs move into detailed physiology, hormonal regulation, nesting behaviour, or comparisons with unrelated capture conditions, without clearly tying these points back to the results from this dataset. Streamlining this material so it focuses on what directly informs interpretation of the new reference intervals would improve clarity.

**Response:** Thank you for this suggestion. As per your feedback, we have revised the manuscript to consolidate the discussion section and overall changed some wording to hopefully make things more understandable to the reader. Given the concerns regarding the discussion, we have also adjusted wording and added in several sentences to lessen the “definitiveness” of the conclusions and emphasize interpretative considerations.

Several detailed physiological explanations seem to follow interpretations from Wallace et al. (2008), but the primary citation (Jackson & Prange, 1979) does not fully support some of the mechanistic conclusions drawn. Re-phrasing these parts more cautiously would help avoid overstating the evidence.

**Response:** Done. We have reworded/added in qualifiers for more cautious interpretation of the results – for example (p. 23, lines 1138-1145): “While the lack of data regarding nesting loggerhead metabolism precludes more direct comparisons, larger size generally correlates with greater total oxygen stores and lower metabolic rates (Wallace et al. 2008). Thus, greater average CCL (a direct correlate of body mass in sea turtles) in nesting green turtles compared to loggerheads could, in part, contribute to differences in aerobic capacity between species. If so, larger loggerheads would then also be expected to have greater total oxygen stores and capacity for aerobic metabolism than smaller loggerheads. This, however, contradicts the positive correlation between lactate and CCL_min_ in nesting loggerheads, which would instead suggest a greater degree of anaerobic metabolism in larger turtles.”

The discussion of “life-stage classes” could also be clearer. Some examples (e.g., entangled turtles, directly captured turtles) describe capture conditions rather than true life stages, and revising this terminology would improve accuracy.

**Response:** Thank you for the suggestion. We have reworded/reorganized the discussion so that this is clearer. The section regarding “life-stage classes” (meaning reproductive nesting female turtles vs non-nesting turtles) and the associated discussion of hormone concentrations implicated in heightened metabolism has been moved forward in the discussion (p. 19, lines 884-892): “While circulating hormone concentrations implicated in heightened metabolism and energy regulation have been evaluated during different stages of the nesting process (epinephrine, norepinephrine, and corticosterone; adrenaline and noradrenaline; aldosterone and thyroxine), and ventilation and metabolic rates have been measured during exercise in nesting green turtles, there has been no direct comparison to non-nesting females to quantify metabolic changes associated with reproduction and long-distance migrations from foraging to nesting areas. Apart from life-stage class, a number of distinct but interrelated factors may contribute to intra-species differences in venous blood gases and lactate, whether compared to non-nesting female turtles or to nesting turtles in other studies.” This is then followed by comparison of levels of activity just prior to capture, then muscle exertion from capture/restraint, which have been differentiated from each other with more direct wording/sentence structure – (p. 20, lines 922-924) “In sea turtles, both type and level of activity (e.g., swimming, diving, digging or egg-laying) just before capture may affect metabolic rate and oxygen consumption, thereby altering acid-base balance.” vs. (p. 21, lines 1002-1004) “Independent of pre-capture activities, blood gases and lactate are possibly most affected by muscle exertion from capture, handling, and/or restraint, due to the high oxygen demand of contracting muscle tissues.”

There are also several long comparisons of blood-gas and lactate values across many unrelated studies. Condensing these comparisons, and linking them more directly to the present dataset, would make the narrative easier to follow. Additionally, the interpretation of lactate differences appears reversed: lower lactate usually reflects less exertion, not more. Revising this would make the physiological interpretation more accurate.

**Response:** Thank you for your suggestion. There have been major edits to the structure and organization of this part of the discussion section to condense comparisons and make things more concise. We have also adjusted the wording regarding the lactate differences as noted since the original wording was confusing (p. 21, lines 1016-1017): “Lower lactate in nesting green turtles in our study compared with studies that used significant restraint may thus be indicative of lesser handling stress.”

The point linking lower lactate to handling stress may also need adjustment. Since lactate mainly reflects muscular exertion, and the turtles were described as not struggling, it may be better to state that the lower values are consistent with minimal exertion and possibly lower handling-related stress, rather than definitive evidence of no stress.

**Response:** Thank you for your suggestion. The wording has been adjusted to make it clearer that turtles were minimally restrained (p. 21, lines 1010-1012): “Turtles in this study were minimally restrained (i.e. minor adjustments of the head) for blood draw compared to routine needs for venipuncture in other settings.” Additionally confusing (p. 21, lines 1016-1017): “Lower lactate in nesting green turtles in our study compared with studies that used significant restraint may thus be indicative of lesser handling stress.”

One practical clarification could also help: blood sampling during oviposition can sometimes cause turtles to pause laying. If this occurred, noting it (and whether it influenced sampling duration or analyte values) would be useful. If it was never observed, a brief statement confirming that sampling did not interrupt oviposition would reassure readers that the behaviour was not disrupted.

**Response:** Done. No turtles paused egg laying during sampling; this has been clarified in the methods section (p. 6-7, lines 207-220): “No turtles were tagged or handled before eggs were laid, and sampling did not disrupt oviposition for any of the turtles in this study.”

Overall, this section would benefit from tightening and focusing on the most relevant physiological context, specifically, the patterns found in this study. A more concise approach will keep the reader centred on the new reference intervals and the key results, without losing important context.

**Response:** Thank you for your constructive feedback! We have reorganized and consolidated the discussion, and overall changed some wording to hopefully make things more understandable to the reader. The headings of the discussion have been removed to assist with consolidation and “flow” of this section. The correlations between blood gases and lactate have now been moved to the first paragraph of the discussion, to introduce the complex interactions of metabolic and respiratory compensatory mechanisms which sea turtles experience during nesting. Next, we move into a discussion of factors that affect acid-base homeostasis and which thus could explain to the reader why blood gas analytes and lactate reference intervals in our study may differ from previously published reference ranges. These include (and have been reorganized in the manuscript accordingly): life-stage class (i.e., actively nesting females with differing hormone concentrations and thus metabolism compared to turtles in non-reproductive life-stage classes), type and level of activity just prior to capture (e.g. swimming, diving, digging, etc), and muscle exertion from the stress of capture/restraint.

CORRELATIONS BETWEEN BLOOD GASES AND LACTATE and SPECIES DIFFERENCES

The discussion around correlations and species differences contains several interpretations that reach beyond what the data can directly support. In both sections, a number of physiological mechanisms are assigned to the observed patterns in lactate and blood gases—such as aerobic capacity, anaerobic thresholds, oxygen storage, metabolic rate, and nesting effort—even though none of these variables were measured in this study. Blood gases and lactate can suggest general acid–base dynamics, but they cannot on their own confirm detailed metabolic strategies, so some of the mechanistic explanations should be framed more cautiously.

**Response:** Thank you for your feedback. We have reorganized, consolidated, and reworded sections of the discussion to improve flow and clarity regarding these concerns to the reader. For example, we have clarified how acid-base homeostasis (i.e., blood gases and lactate) are related to metabolic rates and aerobic limits (p. 19, lines 881-884): “Acid-base homeostasis is significantly impacted by metabolic rates and aerobic limits, which themselves are affected by body size, temperature, activity (e.g., exercise, diving, or muscle exertion during capture and restraint), disease, and hormonal and dietary states.” The discussion regarding metabolic rates and aerobic capacity are presumptive based on prior publications of these data for these or other sea turtle species, as we wish to present the reader with multiple possible interpretative explanations for the differences noted between species. We have also added qualifier words to frame our interpretations more cautiously (e.g., “may” or “likely” p. 22, lines 1062, 1068; p. 24, line 1347, etc).

Similarly, behavioural descriptions (e.g., nest-digging effort, time spent on the beach, frequency of pauses) are used to infer underlying metabolic processes, but these behaviours were not quantified here. Without behavioural or metabolic measurements, it is difficult to link species differences in lactate or pH directly to differences in aerobic capacity or fuel use. Interpretations such as similar lactate indicating higher aerobic capacity in green turtles should therefore be softened, as lactate alone is not a definitive indicator of aerobic strategy.

**Response:** Thank you for these considerations, but we respectfully disagree with this feedback since the behavioral and reproductive aspects are well described in the literature and consistent by species. Therefore, measuring the behavioral variables and differences between species would have been beyond the scope of this study and not needed. We have also clarified how acid-base homeostasis (i.e., blood gases and lactate) are related to metabolic rates and aerobic limits (p. 19, lines 881-884): “Acid-base homeostasis is significantly impacted by metabolic rates and aerobic limits, which themselves are affected by body size, temperature, activity (e.g., exercise, diving, or muscle exertion during capture and restraint), disease, and hormonal and dietary states.” The discussion regarding metabolic rates and aerobic capacity are presumptive based on prior publications of these data for these or other sea turtle species, as we wish to present the reader with multiple possible interpretative explanations for the differences noted between species. As suggested, we have softened the wording to present these conclusions more cautiously.

A few of the citations also appear to be stretched beyond what they support. For example, Jackson & Prange (1979) did not study nesting green turtles, and metabolic scope data from hatchlings cannot be extrapolated to adults during nesting. Some statements linking turtle size, oxygen stores, and metabolic rate may also simplify relationships that were not assessed in this dataset.

**Response:** Thank you for these considerations. Jackson & Prange (1979) studied ventilation, metabolic rate, blood gases and lactate in adult female green turtles nesting at Tortuguero, Costa Rice (6 turtles were samples while actively covering nests, and 5 turtles were captured after nesting for additional study). However, we have removed the comparison to metabolic scope data from hatchlings, as per your suggestion. We have also softened the wording regarding turtle size as it may relate to oxygen stores and metabolic rate, and present these as points for the reader to consider as they interpret the data; for example (p. 23, lines 1138-1143): “While the lack of data regarding nesting loggerhead metabolism precludes more direct comparisons, larger size generally correlates with greater total oxygen stores and lower metabolic rates. Thus, greater average CCL (a direct correlate of body mass in sea turtles) in nesting green turtles compared to loggerheads could, in part, contribute to differences in aerobic capacity between species. If so, larger loggerheads would then also be expected to have greater total oxygen stores and capacity for aerobic metabolism than smaller loggerheads.”

Overall, both sections would benefit from reducing speculative or mechanistic explanations and keeping the focus on what the data clearly show: the observed correlations among analytes and the measured species differences in blood gas and lactate values. Presenting the interpretations more cautiously, and ensuring that all supporting references align directly with the statements made, would strengthen the clarity and accuracy of the discussion.

**Response: Thank you for your suggestions. Overall, we have reorganized, restructured, and consolidated the discussion section, including removal of subheaders, to better integrate and hopefully present a clearer analysis of the data to the reader. As suggested, we have also clarified terminology and edited wording such that the discussion of species differences in regard to metabolic capacity are presented in a more presumptive manner, presenting various points for the reader to consider as they themselves interpret the data, rather than giving definitive conclusions.**

LACTATE METHOD COMPARISONS

The manuscript notes that an i-STAT cartridge costs “~$25 USD” and a Nova Lactate Plus strip costs “~$2 USD.” Because prices vary by region and tend to change over time, it may be clearer to describe these simply as relative consumable costs rather than providing specific dollar amounts.

**Response:** Done. We have removed specific dollar amounts.

Some of the wording around device “validation” may also be a bit strong. The analyses performed constitute a method comparison rather than full analytical validation (e.g., accuracy, precision, repeatability), so a small adjustment in terminology would help keep expectations aligned with what was actually done.

**Response:** Edited. We have changed the wording to “evaluated” instead of “validated” (pg. 25, line 1413).

The detailed temperature-operating ranges for each device may be more specific than necessary unless those limits directly affected data interpretation in this study. More importantly, because temperature corrections were applied to the blood gas values, it would be helpful for the manuscript to report the actual environmental temperatures recorded during sampling. Blood gas and lactate measurements in ectotherms are temperature-sensitive, so noting the temperature range used for corrections would improve clarity for readers.

**Response:** The temperatures were all reported in Table 1 with the reference intervals – ambient temperature (°C) mean +/- SD = 26.1 +/- 1.8, range 21.1 – 28.3°C. The authors do feel that informing the reader of the temperature ranges for each device is useful for those that may be deciding between purchasing one or the other for similar work.

CONCLUSION

A few points in the conclusion could be phrased more cautiously to stay aligned with the data. The study did include a seasonal analysis, but only loggerheads showed significant trends—and these were weak (r² = 0.09–0.21). Green turtle data were limited and uneven across years, so wording such as “across the nesting season” may overstate what can be concluded. A softer phrasing would help.

**Response:** Thank you for the suggestions. We have edited this sentence in the Conclusions accordingly, including the qualification of “across portions of the nesting season” and indicating that the trend toward acidosis is “mild” and “may indicate” differences in physiological strategies between species during nesting (p. 26, lines 1494-1497): “Both loggerheads and green turtles experience complex interactions of metabolic and respiratory compensatory mechanisms, and a trend toward mild lactic acidosis across portions of the nesting season in loggerheads may indicate that physiological strategies to manage periods of high-energy utilization during nesting vary between species.”

Similarly, the suggestion that the two species use different physiological strategies during high-energy nesting is stronger than the results can support, as these mechanisms were not directly measured. Presenting this instead as a possible explanation would be more appropriate. **Response:** Thank you for your feedback. We have included qualifying words such as “mild” and “may indicate” to soften the wording in this sentence (p. 26, lines 1495-1496): “… a trend toward mild lactic acidosis across portions of the nesting season in loggerheads may indicate that physiological strategies to manage periods of high-energy utilization during nesting vary between species.”

The closing section on reference intervals could also be streamlined. Terms like “species-specific,” “cohort-specific,” and “within a given species” overlap and make the message unclear. A simpler statement—e.g., that species-specific RIs will assist future comparisons—would read more cleanly. Additionally, the term “cohort-specific” may not be the best fit here, as it’s not clear what cohort refers to in this context.

**Response:** Thank you for these considerations. The authors respectfully disagree with this assessment. Species-specific means that the reference intervals are specific to each species as opposed to sea turtles or chelonians as a whole. Cohort-specific indicates a specific group within a given species; in this case, nesting turtles (other cohorts would be e.g. non-reproductive foraging turtles which may be male or female). Moreover, the use of “within a given species” underscores the previous sentence about establishing species- and cohort-specific reference intervals; it is meant to tell the reader that, as the data and discussion have shown, there may be variability in blood gases and lactate even within a single species depending on the group (i.e. cohort) that is sampled – e.g. differences in sex, life-stage class, geographical location, activity, disease, hormonal and dietary states, body size, methodology, etc.

Finally, the sentence about identifying stressors or disease isn’t supported by the analyses presented, as the study did not evaluate disease or clinical diagnostic use. Consider softening or removing this unless additional evidence is provided.

**Response:** We agree. Identification of stressors or disease in relation to changes blood gases and lactate is beyond the scope of this study. We included this statement because these data will “help inform” those interpretations. As mentioned in the introduction section, “venous blood gases and lactate are routinely used to evaluate metabolic and respiratory status of stranded turtles…” (which strand and present to rehabilitation facilities due to the underlying stressors or disease).

**Editor Comments**

The manuscript is scientifically sound and clinically useful, but several sentences in the Methods (and to a lesser extent the Introduction and Discussion) are quite awkwardly phrased or dense. I have flagged some examples in the line-by-line section, but have avoided doing full copy-editing. A general language and style review (especially for long, parenthetical sentences and consistency of terminology) would improve readability.

**Response:** Thank you for your constructive feedback! We have revised the manuscript accordingly to address these concerns.
